# Study Insights into Gastrointestinal Cancer through the Gut Microbiota

**DOI:** 10.1155/2019/8721503

**Published:** 2019-06-24

**Authors:** Fanli Kong, Yi Cai

**Affiliations:** School of Life Sciences, Sichuan Agricultural University, Ya'an 625014, China

## Abstract

The gut microbiome in human is recognized as a “microbial organ” for its roles and contributions in regulating the human homeostasis and metabolism. Gastrointestinal (GI) cancers, especially colorectal cancer (CRC), rank as the most common cancer-related deaths worldwide. Evidences have suggested that the disorder of gut microbiota, also named as “dysbiosis,” is related to the development of a variety of diseases such as inflammatory bowel disease (IBD) and the CRC. However, detailed mechanisms between disease and gut microbiota remain largely unknown. This review introduced the correlation between gastrointestinal diseases and the microbiota in human gut from the recent studies, as well as the roles of microbiota in manipulating the CRC and IBDs development, in order to facilitate future studies and to develop novel methods for the precaution, diagnosis, or even cure of gastrointestinal diseases. Additionally, we also elucidated the possibility of probiotics in treatment against CRC.

## 1. Introduction

Gastrointestinal (GI) cancers, especially colon cancer, caused the second highest deaths resulting from cancer in developed countries with more than 4 million new cases arising each year [1]. In pathological diagnosis, the colorectal cancer (CRC) is mainly caused by unhealthy diet, mutation of genes, inflammation, irregular life schedule, and the disorder of microbiota in the gut, and more than 95% of CRC is believed to occur among people who have no predisposition in genes [2]. Since the early phase of the GI diseases is usually asymptomatic, it poses a significant challenge to clinical diagnosis. The trillions of microorganisms inhabited in/on human body are reported to be more and more important as indicators of or contributors to the human health. The so-called “gut microbiota”, for example, are involved in various essential functions including digestion, metabolism, and sustaining the immune system. For the past couple of decades, a considerable amount of studies had demonstrated the correlation of gut microbiota composition and function with atherosclerotic disease [3], metabolic disease [4], and colon cancer [5]. Obesity, for example, is proved to be partly determined by the community structure changes of the gut microbiota. At present, methods of colonoscopy, computed tomography, and blood tests, etc. are widely used for early clinical diagnosis, though the inspection of the gut microbiota is expected to be an alternative way to detect GI cancers early. Nevertheless, most of the researches concentrated on the correlation of altered microbiota and pathophysiology instead of the causative mechanism of the change of microbiome. Although there are significant efforts in improving treatment, such as the development of new drugs, the prognosis for advanced stages of GI disease even the GI cancers is still limited. Consequently, this review mainly elaborates the potential relationship between gut microbiota and the development of GI cancers based on published researches and discusses the influence of major external factors on the constitution of gut microbiota as well as explores the potential of probiotics in the treatment against GI cancers.

## 2. Human Gut Microbiota

The gut microbiota, colonized in gastrointestinal (GI) tract, consists of various microorganisms including bacteria, archaea, viruses, fungi (mostly yeasts), and some eukaryotes. Most of the bacteria belong to the phyla of* Firmicutes* and* Bacteroidetes* accounting for more than 90% in healthy human intestinal tract, followed by* Actinobacteria*,* Proteobacteria*,* Verrucomicrobia*, and* Fusobacteria* [6, 7]. The population of the bacteria in the stomach and small intestine is rather small, while the concentration of microorganisms in the large intestine is significantly higher. Studies showed that there are probably around 1,000-1,150 bacteria existing in human colon [6, 8]. Most of the microbiome show a favorable symbiotic relationship with the host [9, 10]. However, when the microbiome composition and function are perturbed, it will be increasingly linked to various conditions and diseases including cancers, obesity, metabolic diseases, diabetes, allergies, depression, and disorders in the immune system [11, 12]. Meanwhile, a variety of factors including genetics, diet, age, antibiotics, mode of delivery, stress, environmental factors as well as psychological factors can influence the constitution and structure of microbiota in human gut [13].

With the emergence of next-generation sequencing technology, the links between the gut microbiota and its related diseases have been the focus of research. What's more, the microbiome analysis also opens up a new insight into developing biomarkers to diagnose disease and management even at potential therapeutic applications. For example, particular categories of bacteria are involved in the initiation of cancer, e.g.,* Clostridia*,* Streptococcus bovis*,* Helicobacter pylori*, and* Bacteroides, *while other categories of bacteria, e.g.,* Bifidobacterium longum *and* Lactobacillus acidophilus*, can suppress carcinogen and induce colon tumor development, which means the gut microbiota also shows a balance between “detrimental” and “beneficial” bacteria to act on host [14]. However, it is still a big challenge for studying mechanism of gut microbiota in host metabolism, that is because (1) the human gut microbiota is showed high interindividual variability and big complexity, (2) these microorganism interacting with each other is influenced by food inconsistent with the host, and (3) the majority of microbiota are nonsequenced members, which heavily limits interpretation.

## 3. The Main Gastrointestinal Disease and Gut Microbiota

The gut microbiota is highly active and deeply involved in the mucosal immune system and the maintenance of intestinal homeostasis. Not only are various environmental factors such as diet and life style related to IBD and GI cancers, changes of the gut microbiota also lead to IBD and colon cancer. The potential roles of dysbiosis of gut microbiota during the GI diseases focusing on inflammatory bowel diseases and colon cancer are discussed as follows.

### 3.1. The Inflammatory Bowel Diseases (IBD)

IBDs are a category of long-term inflammatory disease of gastrointestinal tract in human, associated with a number of environmental factors. The pathogenesis includes mucosal barrier function and ulcers in patients. Crohn's disease (CD) and ulcerative colitis (UC) are two predominant IBDs conditions that cause the disorder of the epithelial barrier in the intestine as well as the immune system's response to gut microbiome [15]. In general, UC is an inflammatory disease confined to the colonic mucosa, whereas CD has the potential to develop along the entire gastrointestinal tract in small and large intestines. The change of gut microbiota, or dysbiosis, very likely causes IBD in recent researches, but it is still unknown whether the microbiota changes initiate the disease pathogenesis. The immunity to IBD was reported to be strongly determined by polymorphisms in bacterial sensor genes in the host including nucleotide-binding oligomerization domain-containing protein 2 (NOD2), which is also named as caspase recruitment domain-containing protein 15 (CARD15) [16, 17], and toll-like receptor 4 (TLR4) [18]. The* Fusobacterium* species might be related to IBD, such as Crohn's disease and ulcerative colitis (UC). And a study showed that patients who suffer from CD or UC have different community structures of gut microbiome using PCR-based approaches [19, 20] (Table 1). More specifically, by using the next-generation sequencing method, the microbiome was found to be in disorder with higher concentration of* Neisseriaceae, Fusobacteriaceae, Enterobacteriaceae*,* Veillonellaceae*,* Gemellaceae*, and* Pasteurellaceae* and lower concentration of* Bacteroidales, Erysipelotrichaceae*,* Clostridiales, *and* Bifidobacteriaceae* [6, 21, 22]. Moreover, individuals who suffer from chronic IBD have higher possibilities of developing colorectal cancer [23], which accounts for almost 10-15% of their deaths [24, 25].

### 3.2. The Colon/Colorectal Cancer (CRC)

Epidemiologic studies have shown that the cause of colon or colorectal cancer (CRC) includes heredity, diet, environmental factors, life style, microbial exposures, and host immunity [26, 27]. The incidence of CRC mainly occurs in Western countries with high-fat, low-fibre diet (so-called Western diet), seldom in South America, Africa, and Asia, but there's no direct relationship between CRC and the diet. Genetically, the CRC is adenocarcinomas originating in epithelium by DNA damage or chromosomal instability or chronic inflammation [28], and chronic inflammation is widely recognized as a risk factor for CRC initiation and development [29].

Colonic mucosa is a place that regulates the differentiation and proliferation of the epithelial cells, defenses the invasion of pathogenic bacteria, synthesizes the bioactive products as well as fundamental nutrients, and stimulates the immune system [30]. For long, colonic mucosa is believed to be one of the causes of colon tumorigenesis for its constant exposure to the microbiome and/or its metabolites which can cause a long-term inflammatory response in the inflammatory cells. In this case, the colonic microbiota is a potential key factor to CRC [31, 32]. Therefore, the researches on the roles of gut microbiota in the development of tumorigenesis and colon cancer have drawn a considerable amount of attention. Harold et al. proposed a bacterial “driver-passenger model” to show the important roles of microbial involvement in the CRC development [33]. In detail, the “driver” bacteria can initiate the occurrence of CRC, eventually the “passenger” bacteria can replace the “driver” bacteria to promote or stall tumorigenesis. In recent decades, experiments revealed that the concentration of* Fusobacterium nucleatum* in patients who suffer from CRC was significantly higher than that of healthy people, yet the level of* Firmicutes* and* Bacteroidetes* was significantly low in tumors with abundant* Fusobacterium* [34]. The study found the major difference of colon cancer patients from healthy individuals is the high concentration of pathogenic bacteria including* Enterococcus faecalis, Bacteroides fragilis*,* Escherichia coli*, and* Fusobacterium nucleatum* [35]. Boleij et al. showed that the expression of* Bacteroides fragilis* gene was more frequent in CRC patients than that of healthy individuals [36]. Similar finding showed in late-stage cancer patients.

The disorder of gut microbes also leads to the production of harmful metabolites, such as acetaldehyde, secondary bile acid, and glucuronic acid that dramatically influence the development of CRC [37]. In addition, the colonic microbiota may elicit host responses, for example, by stimulating exaggerated immune responses, potentially via Th17 cells, to promote CRC [38]. However, the specific mechanism behind the correlation remains unclear. Recently, researchers used specific bacteria as feed additives in mice to study the interaction between microbiota and the barrier of colonic mucus as well as the interplay of microbe-microbe [39–41]. The induction of* Enterococcus faecalis *shows the genotoxicity of the microbes to colonic epithelial cells [38].* Fusobacterium nucleatum* is the most striking bacteria in CRC. The study showed that it is initially discovered in mouths as a proinflammatory microbe that adheres to the mucosa adherent [42]; subsequently, the* Fusobacterium nucleatum* was also found to promote colon cancer and diseases [43]. Therefore, it is possible to take advantage of* Fusobacterium* to detect colon cancer [44, 45]. Similarly, it is also possible to utilize* Butyricicoccus* and* E. coli* to screen out adenomatous colon mucosa [46, 47]. In Table 2, we showed potential “driver-passenger” bacteria to be used for the detection of CRC.

## 4. Two Main Factors Affecting GI Disease in Gut Microbiota

The human gut microbiota is influenced by a myriad of factors including diet, inflammation, and antibiotics. The links between diet and colon cancer have been plausible for the incidence of colon cancer for a long time. Antibiotics are used to remove or suppress unwanted microorganisms, whereas probiotics/prebiotics can directly introduce the beneficial microorganisms or stimulate the reproduction of microorganisms which are beneficial to the host.

### 4.1. Diet

Diet is a vital factor of modulating the structure of gut microbiota. It determines the prevention and initiation as well as progression of disease or cancer. Studies showed that unhealthy lifestyle, junk food, and malnutrition contribute to 30-40% of various cancers [48]. The diet components, by changing the gut microbiota composition, can change gut homeostasis and modulate the host inflammatory and immune responses. The direct influence on CRC of diet could be related with excessive consumption of fats and proteins, such as Western-style diet, which has been confirmed by Haenszel and coworkers [49]. The gut microbiota of mice that mostly consume standard food displayed changes in the composition of* Bacteroidetes* and* Firmicutes* within a few weeks after switching to a low-fat diet, and the concentration of* Mollicutes*, a species of* Firmicutes*, decreased dramatically [50, 51]. Further study showed that the mice eating high-fat diet and mice eating low-fat diet were different in colon microbiota, which affected the growth of tumor [52]. In conclusion, to a large extent, CRC and the growth of tumor are associated with changes of gut microbiota caused by unhealthy diet [53].

Moreover, a variety of environmental factors including food and medicine directly influence human gut microbiota. Whether the disorder of microbiota causes CRC depends on the antibiotic system's capability to restore the order of gut microbiota and suppress the reproduction of epithelia controlled by host cell cycle regulating genes [54]. As a result, another dimension, antibiotic alternatives, such as pro/prebiotics, has been a new promising approach to study the interaction between food and our gut microbiome.

### 4.2. Pro/Prebiotics

Not only can diet influence the gut microbiota positively, dietary supplement including probiotics, symbiotics, or prebiotics also influence the well-being of host intestinal tract. Probiotics are live microorganisms that alter the gut microbiota positively. Except for enhancing the immune system via the production of anti-inflammatory factors, probiotics also help generate short-chain fatty acids, antioxidant, and anticancer compounds [55]. Despite some disagreements, it is still widely believed that prebiotics and probiotics benefit gut microbiome [56]. In colon cancer, it is proved that probiotic is a new and promising approach to increase colon cancer of chemotherapy effectiveness [57]. Probiotics benefit the host in different ways including colon cancer prevention, colitis reduction, immune system regulation, blood cholesterol reduction, and suppression of pathogenic bacteria [14]. Furthermore, in animal model, Sivan et al. found that* Bifidobacterium longum *and* Bifidobacterium breve* together could improve controlling cancer, reduce tumor development, and increase the anti-PDL1 role in antitumor [58].* Lactobacillus rhamnosus* strain can decrease cellular proliferation and carcinogenesis [59]. These data suggest that it is possible to use pro/prebiotics as an alternative or assistance factors to anticancer since they not only lower the side effect of traditional cancer treatment but also improve the safety of traditional cancer treatment [60, 61]. However, more clinical trials are needed to test and verify the effects of probiotics in the diagnosis, treatment, even ultimately in the prevention of human disease.

## 5. The Methods Used to Study GI Cancers and Microbiota

Early studies evaluating specific microbes mainly focus on species identification or toxin presence for pathogens based on culture-dependent species including* Streptococcus bovis* [62], which is hard to fully evaluate anaerobic constituents. The next-generation sequencing technique, especially when was applied on bacterial 16S rRNA sequencing, has enabled a great deal of clinical studies including identification of the anaerobic genus* Fusobacterium* related to CRC [63]. The entire genome sequences of* Fusobacterium species* have been compared between cases and controls. So, the advance in high-throughput sequencing technologies will facilitate major improvement to understand the microbial ecology and now these biotechnological applications have been widespread in all aspects from personalized medicine to bioenergy [64, 65]. Using the metagenomic method, they derived a positive correlation between the capacity of host glycan degradation and tumor count and a negative relationship between the potential for butyrate production and tumor count, which demonstrated the function of mucus degraders in tumorigenesis [66]. The short-read next-generation sequencing (NGS)-based method, however, is still limited when capturing bacteria. To capture the effect, the whole-genome shotgun sequencing approach or the complete 16S rRNA gene might be essential. Nowadays, various omics fields contribute to the discovery of disease, such as genomics, metagenomics, transcriptomics, proteomics, metabolomics, and phenomics or exposomics, which creates almost infinite pathogenic results to better identify the central regulators. The multiomics integration is possibly curative, which constitutes the foundation of gut disease treatment for individuals.

Furthermore, faecal microbiota transplantation (FMT), administered intragastrically a suspension of fecal samples from a normal individual into a patient intestinal tract to cure the specific disease, is a feasible strategy [67]. However, FMT is still only offering us a modified selective and temporary gut microbiota that depends on both the donor and the recipient to modify the gut microbiota. For patients who suffer from IBD, clinical benefits are still unpredictable. Baxter et al. showed that tumor generation was associated with microbial composition, such as* Bacteroides*,* Parabacteroides*,* Alistipes*, and* Akkermansia*, as well as several members of* Clostridium* group XlVa that prevent the growth of tumors based on faecal microbiota transplantation from healthy individuals and patients who suffer from CRC [66].

## 6. Future Perspective and Conclusions

Although complex processes lead to the development of GI cancers, recent advances have confirmed the importance of microbiota. However, the mechanism of human microbiome in sustaining human health, however, remains unexplained. For example, some conditions, such as IBD, have very robust signatures across populations, while for obesity, the signatures do not apply across other cohorts [68]. So, we must characterize the archetypical organizations of our microbiome in large populations. Whether it is the disorder of microbiome that causes diseases or vice versa, it is certain that microbiome are valuable tools for diagnosis or prognosis, and we should take advantage of microbiome as biomarkers to detect diseases or cancers. Consequently, researchers are studying the mechanism behind the interaction between gut microbiome and disease treatment in order to eventually master the mechanism, which also poses a higher demand for taxonomic level along with various techniques including metabolomics, transcriptomics, and proteomics.

In the future, the gut microbiota may be medically informative. The patients just provide stool samples to send off sequencing and they'll obtain sufficient information as provided by previous colonoscopy biopsy specimens. Besides, the current drug therapy might be replaced by a new strategy that aims at changing the microbiome in the intestine. Moreover, diet treatment and dietary supplement can also be used to keep the balance of microbiota in intestine to prevent or even cure intestinal illness.

In conclusion, this review mostly introduces the correlation between gut microbiota and gastrointestinal disease as well as its potential treatment. There is still a long way to explore before microbiome-based diagnostics become a routine part of clinical care.

## Figures and Tables

**Table 1:** The studies of correlation between human gut microbiota and GI disease.

Feature				Reference
Diversity	Phylum/class	Order/family	Genus/species	
Microbial diversity↓	Bacteroidetes/Firmicutes↓			[19] in CD patients

			Bifidobacteria↓	[20] in UC patients
			Peptostreptococcus↑	

		Neisseriaceae↑ Fusobacteriaceae↑ Enterobacteriaceae↑ Veillonellaceae↑ Gemellaceae↑ Pasteurellaceae↑		[6, 21, 22] in IBD patients

		Bacteroidales↓ Erysipelotrichaceae↓ Clostridiales↓ Bifidobacteriaceae↓		[6, 21, 22] in IBD patients

			Bacteroides fragilis↑	[36] in colon cancer patients

			Enterococcus faecalis↑ Bacteroides fragilis↑ Escherichia coli↑ Fusobacterium nucleatum↑	[34, 35] in colon cancer patients

	Firmicutes↓ Bacteroidetes↓			[34] in colorectal carcinoma

			Parvimonas micra↑	[69]
			Solobacterium moorei↑	

			Peptostreptococcus anaerobius↑	[70] in CRC patients

**Table 2:** The bacteria of “Driver-Passenger” model in CRC.

“Driver-Passenger” model	Microbiota	Mechanism	Ref.
“Driver” bacteria	Enterococcus faecalis	DNA damage	[71, 72]
	Fusobacterium nucleatum	Host immunity	[73]
	Escherichia coli strains	DNA damage	[74]
	Shigella spp.	Early stage of CRC	[75]
	Salmonella spp.		[33]
	Peptostreptococcus anaerobius	Enrich in CRC patient	[70]

Driver or passenger	Gemella Peptostreptococcus Parvimonas	Enrich in CRC patient	[76]

“Passenger” bacteria	Fusobacterium spp	Suppression CRC	[34]
	Streptococcus gallolyticus subsp. gallolyticus	Promotion CRC	[77]
	Clostridium septicum	Promotion CRC	[78]
	Coriobacteriaceae (Slackia and Collinsella spp.)	Suppression CRC	[79]
	Roseburia		
	Faecalibacterium		
